# Fabric Phase Sorptive Extraction: A Paradigm Shift Approach in Analytical and Bioanalytical Sample Preparation

**DOI:** 10.3390/molecules26040865

**Published:** 2021-02-06

**Authors:** Abuzar Kabir, Victoria Samanidou

**Affiliations:** 1 Department of Chemistry and Biochemistry, International Forensic Research Institute, Florida International University, 11200 SW 8th St, Miami, FL 33199, USA; akabir@fiu.edu; 2 Laboratory of Analytical Chemistry, Department of Chemistry, Aristotle University of Thessaloniki, 54124 Thessaloniki, Greece

**Keywords:** extraction, sample preparation, green analytical chemistry, fabric phase sorptive extraction

## Abstract

Fabric phase sorptive extraction (FPSE) is an evolutionary sample preparation approach which was introduced in 2014, meeting all green analytical chemistry (GAC) requirements by implementing a natural or synthetic permeable and flexible fabric substrate to host a chemically coated sol–gel organic–inorganic hybrid sorbent in the form of an ultra-thin coating. This construction results in a versatile, fast, and sensitive micro-extraction device. The user-friendly FPSE membrane allows direct extraction of analytes with no sample modification, thus eliminating/minimizing the sample pre-treatment steps, which are not only time consuming, but are also considered the primary source of major analyte loss. Sol–gel sorbent-coated FPSE membranes possess high chemical, solvent, and thermal stability due to the strong covalent bonding between the fabric substrate and the sol–gel sorbent coating. Subsequent to the extraction on FPSE membrane, a wide range of organic solvents can be used in a small volume to exhaustively back-extract the analytes after FPSE process, leading to a high preconcentration factor. In most cases, no solvent evaporation and sample reconstitution are necessary. In addition to the extensive simplification of the sample preparation workflow, FPSE has also innovatively combined the extraction principle of two major, yet competing sample preparation techniques: solid phase extraction (SPE) with its characteristic exhaustive extraction, and solid phase microextraction (SPME) with its characteristic equilibrium driven extraction mechanism. Furthermore, FPSE has offered the most comprehensive cache of sorbent chemistry by successfully combining almost all of the sorbents traditionally used exclusively in either SPE or in SPME. FPSE is the first sample preparation technique to exploit the substrate surface chemistry that complements the overall selectivity and the extraction efficiency of the device. As such, FPSE indeed represents a paradigm shift approach in analytical/bioanalytical sample preparation. Furthermore, an FPSE membrane can be used as an SPME fiber or as an SPE disk for sample preparation, owing to its special geometric advantage. So far, FPSE has overwhelmingly attracted the interest of the separation scientist community, and many analytical scientists have been developing new methodologies by implementing this cutting-edge technique for the extraction and determination of many analytes at their trace and ultra-trace level concentrations in environmental samples as well as in food, pharmaceutical, and biological samples. FPSE offers a total sample preparation solution by providing neutral, cation exchanger, anion exchanger, mixed mode cation exchanger, mixed mode anion exchanger, zwitterionic, and mixed mode zwitterionic sorbents to deal with any analyte regardless of its polarity, ionic state, or the sample matrix where it resides. Herein we present the theoretical background, synthesis, mechanisms of extraction and desorption, the types of sorbents, and the main applications of FPSE so far according to different sample categories, and to briefly show the progress, advantages, and the main principles of the proposed technique.

## 1. Introduction

When an analytical or bioanalytical chemist is presented with a sample for analysis, regardless of the nature of the sample, a number of important decisions must be made such as which chromatographic/electrophoretic instrument will be used and what the sample preparation strategy would be, among others. Unless the analyst is imposed with some regulatory restrictions, the analyst may independently decide as to whether a solvent based extraction technique (e.g., liquid–liquid extraction, liquid phase microextraction) or a sorbent based extraction technique (e.g., solid phase extraction, solid phase microextraction, stir bar sorptive extraction) [1,2,3] will be deployed. If the goals of the sample preparation are to achieve highly selective extraction of the target analytes as well as to minimize the matrix interference, the obvious choice would be sorbent-based extraction techniques. Subsequently, another major decision point would be whether the sample preparation technique is an exhaustive one as used in solid phase extraction (SPE), or an equilibrium driven one as used in solid phase microextraction (SPME). Both the techniques have some advantages and shortcomings. In addition to the differences in the extraction mechanism, they use almost exclusively two different sets of sorbents (with a few exception). SPME and its different modifications can be deployed in the field, whereas SPE is not generally field deployable. What if an analyst wants to exploit all the advantageous features of both the techniques while minimizing the inherent shortcomings? Keeping this dilemma in mind, Kabir and Furton [4] developed fabric phase sorptive extraction in 2014 as a new generation sample preparation technique that innovatively combines both SPE and SPME in a single sample preparation technology platform. Fabric phase sorptive extraction (FPSE) simultaneously exerts exhaustive extraction mechanism as well as equilibrium driven extraction during the sample preparation process and consequently accomplishes exhaustive or near exhaustive extraction even when the extraction is carried out under equilibrium extraction conditions (e.g., direct immersion extraction). As such, FPSE is neither a new format of SPME nor a new format of SPE, but a true combination of both the techniques.

FPSE has not only combined the extraction mechanisms of SPE and SPME, it has also successfully made available all the sorbents which are exclusively used in either SPE or in SPME. For example, poly(dimethylsiloxane), PDMS, is a popular sorbent coating used in SPME. On the other hand, the C18 phase is predominantly used in SPE. Now, an analyst may use both the sorbents in FPSE.

FPSE is the first sample preparation technology that exploits the surface chemistry of the substrate. In fact, the selectivity and the extraction efficiency of the FPSE membrane originate from the organic polymer, one or more organically modified inorganic precursor and the surface chemistry of the fabric substrate. As such, the selectivity and extraction efficiency of sol–gel PDMS coating on cellulose fabric is substantially different from that of sol–gel PDMS coating on polyester or fiberglass fabric.

FPSE also enjoys the enormous advantages of sol–gel synthesis process that chemically binds the organic polymer/ligand to the substrate using an inorganic/organically modified linker. The chemical bonding between the substrate and the polymer assures very high thermal, solvent, and chemical stability of the FPSE membrane. As a result, the FPSE membranes can be exposed to pH 1–13, as well as to any organic solvent without compromising structural and chemical integrity of the extracting polymer. Sol–gel based chemical coating process provides unprecedented batch-to-batch reproducibility. It is worth mentioning that classical extraction and microextraction techniques often use physical coating processes to immobilize the polymer on the substrate surface, resulting in poor reproducibility, limited range of pH stability, and the tendency to swell when exposed to organic sorbents. Sol–gel-derived sorbents are inherently porous with their characteristic sponge-like porous architecture [5]. As such, the sample matrix can easily permeate through the micro and mesopores of the sol–gel sorbents for rapid analyte–sorbent interaction leading to fast extraction equilibrium.

Due to the open bed, planar geometry, the FPSE membrane can be used in an equilibrium-based extraction mode (as in direct immersion extraction in SPME) or in an exhaustive extraction mode (as an SPE disk). Although, the application potential of FPSE membrane as an SPE disk has not fully explored, Lakade et al. [6] has demonstrated that the FPSE membrane can be used as an SPE disk without compromising the quality of the analytical data.

## 2. Theoretical Background

In its classical operational mode (direct immersion extraction), FPSE mimics the extraction principle of solid phase microextraction (SPME) and similar microextraction techniques, including stir bar sorptive extraction (SBSE) and thin film microextraction (TFME). Extraction of the analyte(s) on the FPSE membrane is primarily governed by the difference in partition coefficient of an analyte between the sample matrix and the FPSE membrane, and the mass transfer of the analyte(s) from the bulk of the sample matrix towards the FPSE membrane continues until an equilibrium is established between the two phases. The mass of the analyte(s) extracted by the FPSE membrane (n), under equilibrium extraction conditions, is proportional to the partition coefficient between the FPSE membrane (which varies with different fabric substrates and the sorbent coatings on the substrate surface) and the sample matrix (K_es_), volume of the extracting phase (V_e_), volume of the sample (V_s_), and the initial concentration of the analyte (C_o_).

The mass of the analyte extracted by the FPSE membrane at equilibrium (n) can be expressed as:

(1) $$n=\frac{\mathrm{KesVeVsCo}}{\mathrm{KesVe}+\mathrm{Vs}}$$

When the sample volume is too large compared to the volume of the extracting sorbent (V_e_ << V_s_),

Equation (1) can be simplified as:

n = K_es_V_e_C_o_ (2)

As can be inferred from Equation (2), the mass of the analyte extracted by the FPSE membrane (n) is directly proportional to the volume of the extracting sorbent and is independent of the sample volume. As such, the value of n can be increased by increasing the volume of the extracting sorbent if the initial concentration of the analyte(s) (C_o_) is kept constant [7].

The extraction efficiency of an FPSE membrane depends on: (a) thermodynamic factors, and (b) kinetic factors [8]. The partition coefficient for an analyte between the FPSE membrane and the sample matrix is a thermodynamic criteria that depends on the material properties of FPSE substrate, coated sorbent, mass of the extracting sorbent, temperature at which the extraction is carried out, and chemical state of analyte in the sample matrix, as well as other factors. However, the most simplistic way to increase the mass of the extracted analyte by the FPSE membrane is to increase the volume of the extracting sorbent.

The kinetic criteria determine the rate at which the equilibrium is reached and can be dramatically improved by maximizing the contact surface between the sample matrix and the extracting phase as well as by applying external energetics (e.g., stirring, sonication, orbital shaking) to diffuse the analyte(s) through the boundary layer between the bulk solution and the extracting phase.

According to the kinetic theory of extraction, increasing the volume of the extracting phase by increasing the thickness of the coated sorbent may lead to unsustainably long extraction equilibrium time as demonstrated in Equation (3).

As the extracting phase in a microextraction technique is generally immobilized on the substrate surface in the form of a thin film, the diffusion of the analyte(s) through the boundary layer regulates the rate of extraction (extraction kinetics). The time required to extract 95% of the equilibrium extraction amount of the analyte t_e,95%_ can be calculated as:

(3) $$\mathrm{te},95\%=\frac{B\delta \mathrm{bKes}}{\mathrm{Ds}}$$

where, b = the thickness of the extracting sorbent; δ = thickness of the boundary layer; K_es_ = distribution constant for the analyte between the extracting sorbent and the sample matrix; Ds = diffusion coefficient of the analyte in the sample matrix; B = geometric factors related to the geometry on which the extracting sorbent is immobilized.

It is evident from Equation (3) that the equilibrium extraction time can be reduced by (i) reducing the coating thickness of the sorbent, (ii) increasing the primary contact surface area of the extracting media (smaller B value), and (iii) increasing the analyte diffusion in the sample matrix by applying external energetics such as magnetic stirring, sonication, orbital shaking, etc.

The rate of extraction in the FPSE membrane, like other microextraction techniques, is not linear. The extraction proceeds very fast in the beginning of the extraction process, and the extraction rate steadily decreases as the extraction progresses towards the equilibrium. The initial rate of extraction ($\frac{\mathrm{dn}}{\mathrm{dt}})$ is directly proportional to the surface area of the extracting phase A, as shown in Equation (4).

(4) $$\frac{\mathrm{dn}}{\mathrm{dt}}=\left(\frac{\mathrm{Ds}\;A}{\delta }\right).\;\mathrm{Co}$$

As such, if one is to increase the sensitivity of the microextraction technique, the volume of the extracting sorbent must be increased. In the same time, in order to reduce the extraction equilibrium time, the primary contact surface area of the extraction device must be augmented.

Fabric phase sorptive extraction has eloquently exploited both the thermodynamic and the kinetic criteria of the microextraction process. It utilizes sorbent loading approximately 40,000× times higher than SPME fiber and 150× times higher than stir bar. Regarding primary contact surface area, the FPSE membrane (in its typical 2.5 cm × 2.0 membrane size) is 50–100 times higher than SPME fiber and 10 times higher than stir bar.

In addition, the sponge-like porous architecture of sol–gel-derived hybrid inorganic–organic sorbent is highly favorable for achieving a fast extraction equilibrium compared to their classical counterparts: highly viscous pristine organic/inorganic polymers traditionally used in most of the microextraction techniques.

## 3. Preparation of Sol–Gel Sorbent Coated FPSE Membranes

Preparation of sol–gel sorbent coated FPSE membrane involves a number of decision points, including:

(1) Selection and pretreatment of the fabric substrate.
(2) Design and preparation of the sol solution for creating the sol–gel sorbent coating on the treated fabric substrate.
(3) Sol–gel sorbent coating process using dip coating technology.
(4) Aging, thermal conditioning, and cleaning of the sol–gel sorbent coated FPSE membrane.
(5) Based on the sample volume, cutting the FPSE membrane into the appropriate size.

It is worth mentioning that steps 1–3 primarily depend on the physicochemical properties of analytes, especially the polarity and molecular state of the analytes.

### 3.1. Selection and Pretreatment of Fabric Substrate

Among all the microextraction techniques, FPSE is the only sample preparation technique that exploits the surface chemistry of the fabric substrate. In general, if the analytes are nonpolar, a hydrophobic substrate such as polyester is the rational choice. When the analytes are polar or medium-polar, a hydrophilic fabric substrate such as 100% cotton cellulose is the judicious selection. Sol–gel sorbents are chemically bonded to the fabric substrate. To ensure this chemical bonding, the fabric substrate should possess abundant surface hydroxyl functional groups. Another important selection criterion for the fabric substrate is its permeability so that the aqueous sample containing the analyte(s) of interest can permeate through the FPSE membrane easily even after creating the sol–gel sorbent coating on the substrate surface. The through pores of the FPSE membrane can extract the analyte(s) almost exhaustively at a short period. The selection of the fabric substrate is followed by the pretreatment of substrate to remove any residual finishing chemicals from the fabric surface. To clean the fabric substrate and to activate surface hydroxyl groups, a fabric treatment protocol has been developed. The protocol can be found elsewhere [7,9].

### 3.2. Design and Preparation of the Sol Solution for Creating the Sol–Gel Sorbent Coating on the Treated Fabric Substrate

The most important step in preparing the FPSE membrane is the design of sol solution. The sol solution for creating sol–gel sorbent coating on the substrate surface consists of (a) one or more inorganic/organically modified sol–gel precursors, (b) a sol–gel active inorganic/organic polymer, (c) a compatible solvent system, (d) an acid catalyst, and water for hydrolysis. Among numerous available organically modified silane precursors, methyl trimethoxysilane (MTMS) is the most commonly used sol–gel precursor. Other popular sol–gel precursors include phenyl trimethoxysilane (PTMS) and 3-aminopropyl trimethoxysilane (3-APTMS).

Commercially available sol–gel active inorganic/organic polymers are abundant in number and many of them are yet to be explored as viable candidates for an FPSE sorbent. Popular polymers used in FPSE include poly(dimethyl siloxane) (PDMS), poly(ethylene glycol) (PEG), poly(tetrahydrofuran) (PTHF), and poly(dimethyl diphenyl siloxane) (PDMDPS).

Among many commercially available acid catalysts (HCl, acetic acid, hydrofluoric acid, trifluoroacetic acid, oxalic acid), trifluoroacetic acid (TFA) is the most commonly used acid catalyst in sol–gel synthesis.

The sol solution for sol–gel sorbent coating on a fabric substrate is generally prepared in an amber reaction vessel (2 oz.) by sequential addition and subsequent vortexing of the sol–gel precursor, solvent, organic/inorganic polymer, acid catalyst, and water. 

A detail account on the potential chemical reactions involved in the sol–gel sorbent coating process can be found elsewhere [5].

The primary criteria for selecting the sol–gel precursor and the inorganic/organic polymer are based on the polarity and functional makeup of the target analytes. Generally speaking, the higher the number of the intermolecular interactions between the FPSE membrane and the target analytes, the higher the extraction efficiency of an FPSE membrane. The overall selectivity and extraction efficiency of a sol–gel sorbent coated FPSE membrane depend combinedly on the surface chemistry of the fabric substrate, the sol–gel precursor, and the inorganic/organic polymer. As such, the selectivity of the pristine polymers such as PDMS and PEG used in SPME and similar microextraction devices are substantially different than that of sol–gel PDMS and sol–gel PEG coated FPSE membranes. Sol–gel sorbents are highly porous and easily accessible for the aqueous/gaseous sample matrices due to their sponge-like porous 3D polymeric network.

### 3.3. Sol–Gel Sorbent Coating Process Using Dip Coating Technology

The sol solution prepared in step 2 is employed in the sol–gel dip coating process. To initiate the coating process, a segment of the pretreated fabric is carefully submerged into the sol solution. The coating process begins as soon as the fabric substrate is introduced into the sol solution. Typically, the sol–gel coating process continues for 12 h at room temperature. Once the predetermined residence in the sol solution is over, the sol solution is discarded from the reaction vessel, and the sol–gel sorbent coated FPSE membrane is air dried for 1 h.

### 3.4. Aging, Thermal Conditioning, and Cleaning of Sol–Gel Sorbent Coated FPSE Membrane

The air-dried sol–gel sorbent coated FPSE membrane is thermally conditioned in a special conditioning device built inside a gas chromatograph (GC) oven under continuous helium gas flow for 24 h. The temperature of the GC oven is set at 50 °C. After conditioning at 50 °C for 24 h, the FPSE membrane undergoes a cleaning protocol established to remove unbonded sol solution ingredients and reaction byproducts. The FPSE membrane cleaning protocol involves rinsing the membrane in methylene chloride: a methanol mixture (50:50 *v*/*v*) under sonication for 1 h. The rinsing solvent mixture is then drained from the rinsing vessel, the FPSE membrane is air dried for 1 h and thermally condition at 50 °C for 24 h under a helium environment. This step completes the sequence of steps involved in creating the sol–gel sorbent coated FPSE membrane. The FPSE membrane is stored in an air-tight container until it is used in fabric phase sorptive extraction.

### 3.5. Cutting the FPSE Membrane into Appropriate Size

Unlike classical microextraction techniques such as SPME, SBSE, and TFME, the membrane size in FPSE is not fixed and can be adjusted based on the analytical need. For a small volume of sample (for example, blood, plasma, saliva), a small FPSE membrane disc (1 cm diameter) can be used. For a larger sample volume (5–20 mL), a larger membrane size (e.g., 2.5 cm × 2.0 cm) is recommended. Although a larger size of an FPSE membrane favors a faster extraction equilibrium due to higher contact surface area, it requires a relatively larger volume of organic solvent for quantitative back-extraction, which may unnecessarily dilute the analytes prior to injection into the chromatographic system. It is important to note that FPSE eliminates the solvent evaporation and sample reconstitution from the sample preparation workflow, an inevitable step in the SPE workflow. As such, the volume of solvent usage in FPSE back-extraction must be kept at its lowest level as possible.

## 4. Mechanism of Extraction in FPSE

Classical microextraction techniques such as SPME, SBSE, and TFME preferentially employ highly viscous pristine polymeric sorbents including PDMS, PEG, and PA, etc., as the extracting phase. During extraction, the analytes are solvated by the extracting polymeric phase. The diffusion coefficient in the highly viscous polymeric coating enables the analytes to penetrate the whole volume of the coating if enough time is allowed. As such, the mass transfer rate as well as the extraction kinetic is relatively slow in the viscous polymeric sorbent coating. When the analytes are heavier (high molar mass), the diffusion into the polymeric extracting phase is even slower. The extraction kinetic can be enhanced by impregnating the viscous polymeric phases with high surface area carbonaceous particulates material such as divinyl benzene (DVB) and Carboxen. These particles act as a bridge inside the liquid polymeric phases and facilitate faster extraction kinetics.

Unlike pristine polymers used in classical microextraction techniques, FPSE utilizes sol–gel sorbent coating technology that chemically binds the organic/inorganic polymer to the fabric substrate via sol–gel precursor as a cross-linker. The resulting sol–gel sorbent is a 3D polymeric network possessing random linkage between the sol–gel precursor and the inorganic/organic polymer. Sol–gel sorbents are inherently porous with sponge-like porous architecture containing numerous mesopores and micropores. During the analyte extraction, the fabric substrate attracts aqueous sample/analytes via its hydrophilic/hydrophobic surface property. As the analytes approach towards the FPSE membrane, multiple intermolecular interactions between the sol–gel sorbent and the analytes come into play, resulting in successful extraction of the analytes into the sol–gel sorbent. The sorbent loading in the FPSE membrane is very high compared to SPME/SBSE/TFME. Due to the high sorbent loading, only a fraction of analyte retention capacity is utilized during the extraction process (even after the exhaustive extraction) as demonstrated by Mesa et al. [10].

## 5. Types of Sorbents in FPSE

Fabric phase sorptive extraction is the only microextraction technique that offers a complete range of sorbent chemistries including polar, medium polar, nonpolar, cation exchanger, anion exchanger, mixed mode, zwitterionic, as well as zwitterionic mixed mode sorbents. Table 1 provides a list of major FPSE sorbent chemistries. It should be noted that all of these sorbents can be coated either on 100% cotton cellulose (hydrophilic) or on fiber glass (neutral) or on polyester (hydrophobic) substrates. 

## 6. FPSE Method Development

**Figure 1:**
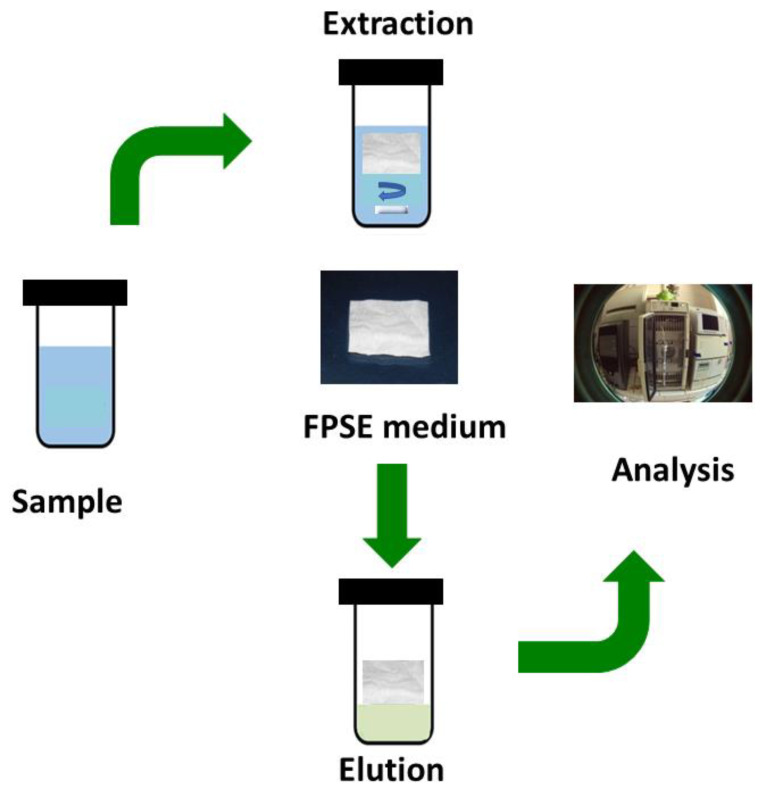
Typical FPSE workflow.

Unlike solid phase microextraction and similar sorbent based microextraction techniques, method development in fabric phase sorptive extraction is simple and straight forward. Figure 1 presents a graphical schematic of a typical FPSE workflow.

**Figure 2:**
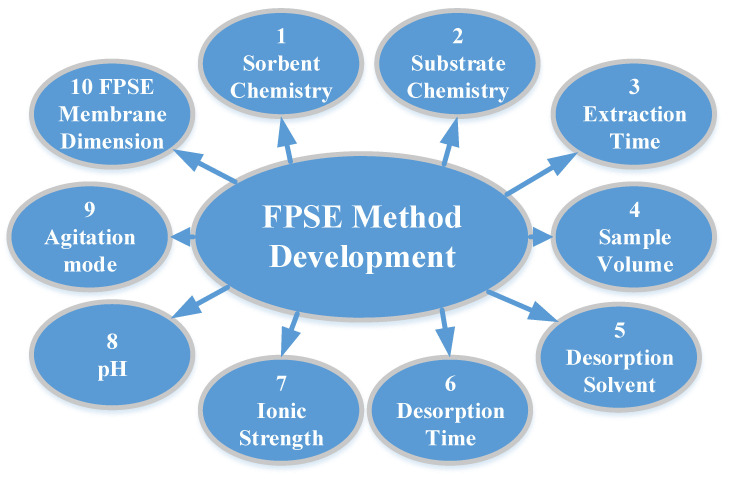
Factors and their relative importance on fabric phase sorptive extraction method development.

FPSE does not require any sample pre-treatment process to reduce/minimize matrix interferents such as filtration, protein precipitation, or centrifugation, and the FPSE membrane can be introduced directly into the sample, regardless of the complexity of the sample. However, the extraction efficiency can be substantially improved when a systematic method development strategy is followed to optimize a number of factors that directly impact on the overall extraction efficiency of the FPSE membrane. The factors are presented in Figure 2 with their relative significance. As such, an analyst may decide which factor(s) should be given more attention during the method development exercises. The factors include:

(i) Sorbent chemistry;
(ii) Substrate surface chemistry;
(iii) Extraction equilibrium time;
(iv) Sample volume;
(v) Desorption time;
(vi) Desorption solvent;
(vii) Ionic strength;
(viii) Sample pH;
(ix) Agitation mode;
(x) FPSE membrane dimension.

FPSE method development exercises can be carried out using a conventional one-factor-at-a-time (One FAT) approach or using a chemometric design of experiment approach. The later approach is the more scientific and green approach, as it provides deep insight about the overall extraction process and sheds light as to whether different factors interact with each other or not. A screening design can be carried out to select factors with the most influence on the overall extraction efficiency. Subsequently, a response surface model (RSM) design can be employed to find the optimum levels of the most influential factors.

### 6.1. Selection of FPSE Sorbent Chemistry

As can be seen in Table 1, FPSE offers a broad range of sorbents spanning from nonpolar, to medium polar, to polar, to ionized, to mixed mode, and to zwitterionic. As such, it is practically impossible for one to determine the most efficient sorbent by real experimentation. As such, for the first time, a new approach for selecting FPSE sorbent chemistry has been developed based on an absolute recovery percentage calculator that utilizes the logKow of an analyte to predict an estimated absolute recovery of an analyte for a given FPSE sorbent chemistry. For example, the absolute recovery on sol–gel Carbowax 20M (sol-gel CW 20M) sorbent coated on 100% cotton cellulose fabric can be expressed as:

Absolute Recovery % = 4.2977487 + 22.823041 × Log Kow − 3.1343544 × (Log Kow − 2.737)^2^

This equation is valid for any analyte possessing a logKow value between 0.3 and 5.07, and majority of the analytes we generally encounter fall in this range. During the FPSE method development exercises, it is recommended that an analyst select the 3 best FPSE membranes, and subsequently determine the best FPSE membrane by exposing them under identical FPSE conditions. A good starting time can be:

FPSE membrane size: 2.5 cm × 2.0 cm;

Sample volume: 10 mL;

Analyte concentration: 1 µg/mL;

Extraction time: 1 h;

Stirring speed: 800 rpm;

Desorption solvent: methanol;

Desorption solvent volume: 500 µL;

Desorption time: 10 min.

The prepared sample can be injected into a gas chromatograph or high-performance liquid chromatograph to obtain the chromatographic signal area for an analyte or group of analytes.

Absolute recovery calculations for major FPSE sorbent chemistries are presented in Table 2.

The predicted absolute recovery values often corroborate with the actual recovery values obtained from real experimentation, as demonstrated by several researchers [11,12]. It is important to note that this model was developed using analyte solution in deionized water. When the sample matrix contains too many matrix interferents, substantial deviation from the expected recovery of the analyte may be observed [13].

### 6.2. Selection of FPSE Substrate

FPSE is the only microextraction technique that exploits the substrate surface chemistry to compliment to the overall selectivity and the extraction efficiency of an FPSE membrane. The surface property of the fabric substrate substantially impacts on the selectivity and extraction efficiency of an FPSE membrane. The dependence of the analytes’ polarity (logKow) on the nature of different fabric substrates has been estimated using a compound mixture consisting of furfural alcohol (FA, logKow 0.3), piperonal (PIP, logKow 1.05), phenol (PHE, logKow 1.5), benzodioxole (BDO, logKow 2.08), 4-nitrotoluene (4NT, logKow 2.45), 9-anthracene methanol (9AM, logKow 3.04), 1,2,45-tetramethyl benzene (TMB, logKow 4.0), triclosan (TCL, logKow 4.53), and diethylstilbestrol (DES, logKow 5.07). As can be seen from Table 3, for the sol–gel PTHF sorbent, cellulose fabric is favored for polar analytes extraction, whereas fiber glass fabric is suitable for nonpolar analyte extraction. Between sol–gel PDMDPS sorbents coated on polyester fabric and cellulose fabric, polyester is better for nonpolar analyte extraction. Since PDMDPS is a nonpolar polymer, extraction of polar analytes is not favored in either polyester fabric or in cellulose fabric. As expected, the organic/inorganic polymer plays the most significant role in the overall selectivity and extraction efficiency of FPSE membrane. However, the role of the fabric substrate cannot be ignored. 

### 6.3. Optimization of Extraction Equilibrium Time

Extraction efficiency is one of the most important factors that influence the extraction efficiency of an FPSE membrane. Generally, extraction efficiency is verified between 0 and 60 min, when most of the analytes reach the plateau of the extraction kinetic curve, and exposing the FPSE membrane longer than this time period does not yield any improvement in the extraction efficiency of an analyte in a given FPSE membrane. In some cases, when a high mass of matrix interferents are present in the sample matrix, as in the case of an environmental or biological sample, longer extraction equilibrium time may be observed. 

### 6.4. Optimization of Sample Volume

Sample volume requirement in FPSE is flexible and depends on the availability and nature of the sample. For a smaller sample volume, a smaller FPSE membrane size can be used. If the sample is freely available, a larger FPSE membrane size (e.g., 2.5 cm × 2.0 cm) can be used, and a sample volume from 10 mL to 30 mL may be systematically investigated to determine the optimum sample volume.

### 6.5. Optimization of Desorption Solvent

Due to the strong chemical bonding between the fabric substrate and the sol–gel sorbent coating, an FPSE membrane can be exposed to any organic solvent for quantitative back-extraction of the analytes after the extraction process. As such, a single solvent or a mixture of solvents can be used to efficiently back-extract the analytes. The solvent or solvent system (mixture of multiple solvents) should be optimized to ensure quantitative back-extraction of the extracted analytes.

### 6.6. Optimization of Desorption Time

Since the sol–gel sorbents are inherently porous with sponge-like morphology, the diffusion of the solvent during solvent mediated back-extraction does not need any external energetic stimulus such as magnetic stirring. However, it is imperative to allow adequate time for the solvent to exhaustively scavenge the extracted analytes from the sol–gel sorbents. Most researchers have reported 5 min as the optimum desorption time, although in some cases 7.5 min or 10 min as the optimum desorption times are not unusual. For method development, a time range between 0 and 10 min can be investigated.

### 6.7. Optimization of Ionic Strength of the Sample Matrix

Ionic strength of the sample matrix can be increased by the addition of NaCl or another suitable salt to the sample to compel polar analytes out of the aqueous solution and become available for being extracted into the FPSE membrane. The optimum salt concentration can be determined by monitoring the increase in the extraction efficiency with the concentration of salt in the solution.

### 6.8. Optimization of pH of the Sample Matrix

When acidic or basic analytes are extracted on a neutral FPSE membrane, pH adjustment of the sample matrix may be used to force the analytes to remain in their neutral state so that the neutral FPSE membrane can maximize its extraction efficiency under the given extraction conditions. It is a cumbersome process and requires obtaining an optimum matrix pH value via a series of experiments. In order to eliminate this cumbersome drill, a mixed mode sorbent coated FPSE membrane can be used. 

### 6.9. Optimization of Sample Matrix Agitation

Extraction kinetics can be expedited by applying external stimuli such as magnetic stirring, ultra-sonication, or orbital shaking during the FPSE process. The optimum stirring speed should be established experimentally during the FPSE method development.

### 6.10. Selection of FPSE Membrane Size

FPSE is the only microextraction technique that allows the analyst to determine the size of the FPSE membrane. Although the typical size for a small volume of sample is a 1 cm diameter disc, or a 2.5 cm × 2.0 cm rectangular block for a larger sample volume, the analyst may use any size of the FPSE membrane depending on the analytical need.

## 7. Applications

All developed methodologies reported in the literature since 2014 are briefly described below, showing the wide range of applicability of fabric phase sorptive extraction in terms of sample matrix and analyte diversity.

Many research groups all over the world have adopted this innovative sample preparation approach and have developed new analytical strategies to deal with significant analytical problems encountered in virtually all facets of analytical fields.

Kumar et al. in 2014 were the first group to implement fabric phase sorptive extraction (FPSE) in the development of a simple, fast, and sensitive analytical method using a sol–gel poly(tetrahydrofuran) (sol–gel PTHF) coated FPSE membrane for the quantification of endocrine disrupting chemicals (EDCs), including 17α-ethinyl estradiol (EE2), β-estradiol (E2) and bisphenol A (BPA). Analysis was performed by high performance liquid chromatography with fluorescence detection (HPLC-FLD). In their work, the authors have investigated and optimized various factors that influence the efficiency of FPSE technique. The developed method was applied successfully for the analysis of the examined estrogen molecules in urine and various kinds of aqueous samples with good reported recoveries, i.e., 96–98% for drinking water, 94–95% for ground water, 92–94% for river water, and 90–91% for urine samples, while lower detection limits of BPA, E2, and EE2 over previously reported methods were achieved within the range of 20 to 42 pg/mL. Linearity, precision, and accuracy results proved that the developed method is rapid, precise, reproducible, and sensitive for the determination of estrogens in urine and aqueous samples [9]. 

A year later, in 2015, Roldán-Pijuán et al. [14] presented for the first time a novel technique: the approach of stir fabric phase sorptive extraction (SFPSE), which integrates sol–gel hybrid organic–inorganic coated fabric phase sorptive extraction media with a magnetic stirring mechanism. Two flexible fabric substrates, namely cellulose and polyester, were utilized as the host matrix for three different sorbents, e.g., sol–gel poly(tetrahydrofuran) (sol–gel PTHF), sol–gel poly(ethylene glycol) (sol–gel PEG), and sol–gel poly(dimethyldiphenylsiloxane) (sol–gel PDMDPS). Triazine herbicides were selected as model compounds to evaluate the operational performance of this unique microextraction device. The factors affecting the extraction efficiency of SFPSE have been investigated, and the optimal extraction conditions using sol–gel PEG coated SFPSE device in combination with UPLC-DAD yielded limits of quantification (LOQs) for the seven triazine herbicides in the range of 0.26–1.50 μg/L, while the hyphenation with LC-MS/MS allowed the improvement of the method sensitivity to the range of 0.015 μg/L to 0.026 μg/L. Enrichment factors between 444 and 1411 were achieved. The developed method was finally applied for the determination of selected triazine herbicides from three river water samples. Relative recoveries of the target analytes, in the range of 75% to 126%, were found to be satisfactory, while absolute extraction recoveries were in the range of 22.2–70.5%. 

In the same year, Montesdeoca-Esponda et al. [15] developed a fast and sensitive sample preparation methodology using fabric phase sorptive extraction followed by ultra-high-performance liquid chromatography and tandem mass spectrometry detection for the determination of a group of compounds added to sunscreens and other personal care products which may present detrimental effects to aquatic ecosystems, i.e., benzotriazole UV stabilizer compounds in aqueous samples. In their work, the authors optimized the extraction of seven benzotriazole UV filters in terms of several parameters influencing the extraction process, such as sorbent chemistry selection, extraction time, back-extraction solvent, back-extraction time, and the impact of ionic strength. Under the optimized conditions, which included polyester fabric that was used as the substrate for sol–gel PDMDPS coating, FPSE provided enrichment factors of 10 times with detection limits ranging from 6.01 to 60.7 ng L^−1^. Ultra-high-performance liquid chromatography and tandem mass spectrometry detection were used for the determination of target analytes in sewage samples from wastewater treatment plants with different purification processes of Gran Canaria Island (Spain). 

No expensive commercial supplies or instrument were needed. Thus, FPSE was proved to be a cost-effective alternative to other expensive extraction and microextraction methods for the target analytes. 

Kumar et al. in 2015 [12] developed and validated a novel analytical method for the quantification of endocrine-disruptor alkyl phenols, namely: 4-tert-butylphenol, 4-sec-butylphenol, 4-tert-amylphenol, and 4-cumylphenol, in aqueous and soil samples. Analysis was subsequently performed by high-performance liquid chromatography with ultraviolet detection. Various parameters influencing the fabric phase sorptive extraction performance, such as extraction time, eluting solvent, elution time, and pH of the sample matrix, were optimized. Sol–gel PTHF coated FPSE media on cellulose substrate was proved to show the best extraction efficiency with methanol as the extraction solvent, yielding recovery rates of 74.0, 75.6, 78.0, and 78.3 for 4-tert-butylphenol, 4-sec-butylphenol, 4-tert-amylphenol, and 4-cumylphenol, respectively. Optimum conditions offer high preconcentration and enrichment factors and significantly reduced sample preparation time. The limits of detection ranged from 0.161 to 0.192 ng/mL, and the method was successfully applied for the recovery of alkyl phenols from spiked ground water, river water, and treated water obtained from a sewage treatment plant, and a soil and sludge sample. The method reduces the use of organic solvents substantially, meeting the criteria of the green analytical chemistry principle. 

Racamonde et al. in 2015 [16] investigated the use of FPSE for the determination of four nonsteroidal anti-inflammatory drugs (ibuprofen, naproxen, ketoprofen and diclofenac) in environmental water samples prior to their determination by gas chromatography mass spectrometry. Various factors affecting FPSE, namely: sorbent chemistry, matrix pH, and ionic strength, were investigated using a mixed level factorial design (31 × 22), while other important parameters, e.g., sample volume, extraction kinetics, desorption time, and volume, were also optimized. Three different FPSE sorbent chemistries, sol–gel PDMDPS, sol–gel PTHF, and sol–gel PEG, were investigated. Sol–gel PEG coatings on the cellulose substrate with ethyl acetate as the eluent proved to provide optimal operational conditions, leading to the limits of detection (S/N = 3) in the range of 0.8–5 ng L^−1^. The enrichment factors ranged from 162 to 418, while absolute extraction efficiencies varied from 27% to 70%. Satisfactory relative recoveries within the range 82–116% demonstrated that the proposed method can be readily applied to routine environmental pollution monitoring. Actually, the proposed method was successfully applied to the analysis of examined analytes in two influent and effluent samples from a wastewater treatment plant and two river water samples in Spain. 

Compared to other sorptive microextraction techniques, FPSE showed many benefits such as simplicity in device fabrication, low cost, high enrichment factors and faster extraction equilibrium. 

Samanidou et al. in 2015 [17] applied FPSE to develop a simple, sensitive, reliable, and fast analytical methods for the simultaneous determination of residual highly polar amphenicol antibiotics (amphenicols) in raw milk, followed by high-performance liquid chromatography–diode array analysis. A highly polar polymer coated FPSE membrane using short-chain poly (ethylene glycol) (sol–gel PEG) was used. The intense affinity of amphenicols towards the strongly polar sol–gel PEG-coated FPSE device yielded absolute recovery of the selected antibiotics residues in the range of 44% for thiamphenicol, 66.4% for florfenicol, and 81.4% for chloramphenicol. The developed method was validated in terms of sensitivity, linearity, accuracy, precision, and selectivity according to European Decision 657/2002/EC. The decision limit (CCα) values obtained were 52.49 μg kg^−1^ for thiamphenicol, 55.23 μg kg^−1^ for florfenicol, and 53.8 μg kg^−1^ for chloramphenicol, while the corresponding results for detection capability (CCβ) were 56.8 μg kg^−1^, 58.99 μg kg^−1^, and 55.9 μg kg^−1^, respectively.

Lakade et al. in 2015 [18] proposed the use of FPSE applying different coating chemistries, namely: nonpolar sol–gel PDMDPS, medium polar sol–gel PTHF, and polar sol–gel poly(ethylene glycol)-block-poly(propylene glycol)-block-poly(ethylene glycol) (sol–gel PEG-PPG-PEG), and sol–gel Carbowax 20M (sol–gel CW 20M) to the extraction of a group of pharmaceuticals and personal care products (PPCPs) with a wide range of polarity from environmental aqueous samples. Several factors influencing FPSE, such as sample pH, stirring speed, addition of salt, extraction time, sample volume, elution solvent, and desorption time, were investigated and optimized for each sorbent coated FPSE membrane. Optimum conditions included the FPSE membrane coated with sol–gel CW 20M that provided the highest absolute recoveries (77–85%) for most of the analytes, except for the most polar ones. All examined sorbents offered better recovery compared to the commercially available coating for stir bar sorptive extraction based on ethylene glycol/silicone (EG/Silicone). The method based on FPSE with sol–gel CW 20M membrane and liquid chromatography-(electrospray ionization) tandem mass spectrometry (LC-(ESI) MS/MS) was applied to environmental water samples. Good apparent recoveries (41–80%) and detection limits (1–50 ng L^−1^) were achieved. 

One year later, in 2016, Anthemidis et al. [19] developed a novel flow injection fabric disk sorptive extraction (FI-FDSE) system for the automated determination of trace metals. The platform was based on a mini-column packed with sol–gel coated fabric membrane in the form of disks, incorporated into an on-line solid-phase extraction system, coupled with flame atomic absorption spectrometry (FAAS). This configuration resulted in high loading flow rates and shorter analytical cycles due to the minor observed backpressure. The potentials of this technique were demonstrated for trace lead and cadmium determination in environmental water samples. Various sol–gel coated FPSE media were investigated. The on-line formed complex of metal with ammonium pyrrolidine dithiocarbamate (APDC) was retained onto the fabric surface. Among the examined sol–gel coated FPSE membranes, sol–gel PDMDPS coated membrane provided the best extraction sensitivity and excellent reproducibility due to its hydrophobic nature similar to that of metal-APDC complex. The analytes were subsequently eluted by methyl isobutyl ketone (MIBK) prior to atomization. Optimum parameters included 90 s preconcentration time, with a sampling frequency of 30 h^−1^, and thus enrichment factors of 140 and 38 and detection limits of 1.8 and 0.4 μg L^−1^ were achieved for lead and cadmium. 

Huang et al. in 2016 [20] proposed the use of the cellulose fabric as the host matrix, for three extraction sorbents, namely: sol–gel PTHF, sol–gel PEG, and sol–gel PDMDPS, which were prepared on the surface of the cellulose fabric. Two extraction techniques have been proposed. The first one included stir bar fabric phase sorptive extraction (stir bar-FPSE), and the second one magnetic stir fabric phase sorptive extraction (magnetic stir-FPSE), both allowing stirring of fabric phase sorbent during every step of the extraction process. Three brominated flame retardants (BFRs) [tetrabromobisphenol A (TBBPA), tetrabromobisphenol A bisallylether (TBBPA-BAE), tetrabromobisphenol A bis(2,3-dibromopropyl)ether (TBBPA-BDBPE)] were selected as model analytes for the practical evaluation of the two proposed techniques using high-performance liquid chromatography (HPLC). Several experimental conditions which mainly affect the extraction process such as the type of fabric phase, extraction time, the amount of salt, and elution conditions were studied and optimized. Both techniques possessed high extraction capability and fast extraction equilibrium as a result of the large sorbent loading capacity and unique stirring performance. High recoveries (90–99%) and low limits of detection (LODs) (0.01–0.05 μg.L^−1^) were achieved using the optimized conditions. The results were promising, and the methods were shown to be practical for monitoring of hazardous pollutants in the water sample, meeting green analytical chemistry requirements, mainly due to low solvent consumption. 

Guedes-Alonso et al. in 2016 [21] in their study proposed an extraction method based on sorptive fabric phase coupled to ultra-high-performance liquid chromatography tandem mass spectrometry detection (FPSE-UHPLC-MS/MS) for the determination of four progestogens and six androgens in environmental and biological samples. These analytes consist two important groups of endocrine disrupting compounds (EDCs) which may have severe harmful impact on aquatic biota, even at very low concentrations. All the experimental parameters involved in the extraction, such as sample volume, extraction and desorption times, desorption solvent volume, and sample pH values have been optimized. Sol–gel PTHF coated FPSE and analyte desorption with methanol proved to show the best results. The developed method showed satisfactory limits of detection (between 1.7 and 264 ng L^−1^), and good recoveries. The applicability of the method was examined by its use of the analysis of tap water, wastewater treated with different processing technologies, and urine samples. The concentrations of the detected hormones ranged from 28.3 to 227.3 ng L^−1^ in water samples and from 1.1 to 3.7 μg L^−1^ in urine samples. The method showed significant benefits such as minimum usage of organic solvents, short extraction times, small sample volumes, and high analyte preconcentration factors. 

Karageorgou et al. in 2016 [22] used FPSE for the determination of sulfonamides residues in milk using a highly polar sol–gel PEG coated membrane. The developed HPLC method was validated according to the European Union Decision 2002/657/EC. Due to the low organic solvent consumption, the FPSE-based method meets all green analytical chemistry (GAC) criteria. The decision limit (CC_α_) values were 116.5 μg kg^−1^ for sulfamethazine, 114.4 μg kg^−1^ for sulfisoxazole, and 94.7 μg kg^−1^ for sulfadimethoxine, whereas the corresponding results for detection capability (CC_β_) were 120.4 μg kg^−1^ for sulfamethazine, 118.5 μg kg^−1^ for sulfisoxazole, and 104.1 μg kg^−1^ for sulfadimethoxine. 

Samanidou et al. in 2016 [13] evaluated the application of FPSE for the extraction of benzodiazepines from human blood serum. Benzodiazepines were selected as model analytes because they represent one of the most widely used therapeutic drugs in psychiatry and are also amongst the most frequently encountered drugs in forensic toxicology. FPSE was performed using cellulose fabric extraction media coated with sol–gel PEG. Absolute recovery values in the equilibrium state for the examined benzodiazepines were found to be 27% for bromazepam, 63% for lorazepam, 42% for diazepam, and 39% for alprazolam. 

Lakade et al. in 2016 [6] described the use of a new extraction approach based on fabric phase sorptive extraction (FPSE). This new mode proposes the extraction of the analytes in dynamic mode in order to reduce the extraction time. Dynamic fabric phase sorptive extraction (DFPSE) was applied using sol–gel Carbowax 20M material, followed by liquid chromatography–tandem mass spectrometry. This approach was evaluated for the extraction of a group of pharmaceuticals and personal care products (PPCPs) from environmental water samples. Different experimental parameters affecting the extraction were investigated and optimized. Best performance was achieved using ethyl acetate as elution solvent. Recovery rates were higher than 60% for most of the compounds, with the exception of the most polar ones (between 8% and 38%). The analytical method was validated and applied to river water, effluent and influent wastewater, and good performance was obtained. The analysis of samples revealed the presence of some PPCPs at low ng L^−1^ concentrations. 

Aznar et al. in 2016 [23] in order to investigate the migration of additives added to food packaging materials to food in contact with them during storage and shelf life, developed a novel simple, fast, and sensitive analyte extraction method based on fabric phase sorptive extraction (FPSE), followed by analysis using ultra-high performance liquid chromatography and mass spectrometry detection (UPLC-MS). The method was applied to the analysis of 18 common non-volatile plastic additives. Three FPSE media coated with different sol–gel sorbents characterized by different polarities, including sol–gel poly(dimethyl siloxane) (sol–gel PDMS), sol–gel PEG, and sol–gel PTHF, were investigated, and all showed very satisfactory results. Analytes with low logP values (polar analytes) showed higher enrichment factors (EFs), especially with sol–gel PTHF and sol–gel PEG membrane. For compounds with high logP values (nonpolar compounds), the use of sol–gel PDMS improved the enrichment capacity. 

For compounds with low logP values (logP < 5), sol–gel PEG coated FPSE media showed higher enrichment factors. Sol–gel PTHF coated FPSE media, with an intermediate polarity, showed the best EFs values. 

Ten compounds obtained enrichment factors above 3 with sol–gel PTHF coated FPSE membrane, whereas for sol–gel PDMS or sol–gel PEG, only six compounds were above this value. 

Acetonitrile showed best desorption efficiency yielding recoveries over 70% for 13 out of 18 selected compounds in all FPSE media. 

Alcudia Leon et al. in 2017 [24] presented a novel sampling device that integrates air sampling and preconcentration based on fabric phase sorptive extraction principles. The determination of the main components of the sexual pheromone of *Tuta absoluta* [(3E,8Z,11Z)-tetradecatrien-1-yl acetate and (3E,8Z)-tetradecadien-1-yl acetate] traces in environmental air in tomato crops has been selected as a model system. A laboratory-built unit made up of commercial brass elements as a holder of the sol–gel coated fabric extracting phase was designed and optimized. The unit proved to efficiently work under sampling and analysis modes which eliminated any need for sorptive phase manipulation prior to instrumental analysis. In the sampling mode, the unit is connected to a sampling pump to pass the air through the sorptive phase under controlled flowrate. In the analysis mode, the unit is placed in the gas chromatograph autosampler without any instrumental modification, thus eliminating the risk of cross contamination between sampling and analysis. The limits of detection for both compounds resulted to be 1.6 μg and 0.8 μg.

Three different fabric phases coated with sol–gel PEG, sol–gel PTHF, and sol–gel PDMDPS were evaluated for the extraction of two sexual pheromones components from a gaseous standard. The results indicated that the sol–gel PDMDPS proved to show optimum results. In fact, the results confirmed the expected behavior considering the high hydrophobicity (log K_o/w_ are 5.76 and 6.28 for component A and B, respectively) of the target compounds.

Locatelli et al. in 2017 [25] developed a fabric phase sorptive extraction high-performance liquid chromatography-photodiode array detection (FPSE-HPLC-PDA) method for the simultaneous extraction and analysis of twelve azole antimicrobial drug residues (i.e., ketoconazole, terconazole, voriconazole, bifonazole, clotrimazole, tioconazole, econazole, butoconazole, miconazole, posaconazole, ravuconazole, and itraconazole) in human plasma and urine samples. The limit of quantification of the FPSE-HPLC-PDA method was found as 0.1 μg/mL, and good linearity was observed up to a concentration of 8 μg/mL. The performance of the developed method was investigated on real samples from healthy volunteers after a single dose administration of itraconazole and miconazole. The method proved to be a rapid and robust green analytical tool for clinical and pharmaceutical applications.

Three different FPSE membranes were investigated: sol–gel silica Carbowax^®^ 20 M (sol–gel CW20 M) with 8.63 mg/cm^2^ sorbent loading, sol–gel polydimethylsiloxane (sol–gel PDMS) with a sorbent loading of 4.56 mg/cm^2^, sol–gel caprolactone-dimethylsiloxane-caprolactone (sol–gel CAP-DMS-CAP) with a sorbent loading of 6.14 mg/cm^2^, while optimal effectiveness was observed by sol–gel Carbowax^®^ 20 M and methanol as elution solvent.

Heena et al. in 2017 [26] developed a method for the determination of Co(II), Ni(II), and Pd(II) in aqueous samples using fabric phase sorptive extraction high-performance liquid chromatography-UV detection (FPSE-HPLC-UV). A preconcentration step was necessary due to the trace level concentrations of these elements in aqueous samples. Sol–gel polytetrahydrofuran nanocomposite was selected as the optimum sorbent. The limit of detection for Co(II), Ni(II), and Pd(II) morpholino dithiocarbamate complexes were found at much lower concentration levels as compared to earlier reported data with excellent reproducibility. The new FPSE-HPLC-UV method can be used for routine determination of these metal species in various aqueous environmental samples and in different alloys.

Kazantzi and Anthemidis in 2017 [27] developed a novel flow injection on-line fiber fabric sorptive extraction (FI-FFSE) platform, taking advantage of the benefits of the FPSE technique in automatic mode. A microcolumn packed with a sol–gel coated fiber fabric medium, the poly(dimethylsiloxane) (sol–gel PDMS), incorporated into a FI-SPE system, was presented. The low backpressure in this configuration results in high loading flow rates and shorter analytical cycles. The on-line formed complex of metal with sodium diethyl dithiocarbamate (DDTC) is retained onto the fabric surface, while analytes are eluted by methyl isobutyl ketone (MIBK) prior to atomization. For 90 s preconcentration time, enrichment factors of 165 and 43 and detection limits (3 s) of 1.6 and 0.3 µg L^−1^ were achieved for lead and cadmium determination, respectively, with a sampling frequency of 30 h^−1^. The developed method has been successfully applied to the on-line lead and cadmium determination by FAAS in energy and refreshment drinks.

In 2017, Samanidou et al. [28] evaluated fabric phase sorptive extraction (FPSE) as a simple and rapid strategy for the extraction of four penicillin antibiotic residues (benzylpenicillin, cloxacillin, dicloxacillin, and oxacillin) from cows’ milk, without prior protein precipitation. Time-consuming solvent evaporation and reconstitution steps were eliminated successfully from the sample preparation workflow. Short-chain poly(ethylene glycol) provided optimum extraction sensitivity for the selected penicillins, which were analyzed using a Reversed Phase (RP) HPLC method, validated according to the European Decision 657/2002/EC. The limits of quantitation achieved were a similar order of magnitude with those reported in the literature (with the exception of benzylpenicillin) and less than the maximum residue limits (MRL) set by European legislation.

Saini et al. in 2017 [29] applied FPSE to the trace-level determination of four selected polycyclic aromatic hydrocarbons (PAHs), namely: fluoranthene, phenanthrene, anthracene, and pyrene, in environmental water samples using a nonpolar sol–gel C18 coated FPSE media. Extraction efficiency was optimized. Limits of detection (LODs) and quantification (LOQs) were found to be at pg/mL levels: 0.1–1 pg/mL and 0.3–3 pg/mL, respectively. Average absolute recovery rates were in the range of 88.1–90.5%. The applicability of the developed FPSE-HPLC-FLD protocol was proved by the analysis of eight environmental water samples and proved to be simple, green, fast, and cost effective, with adequate sensitivity, and thus it can be applied for routine monitoring of water quality and safety.

Samanidou et al. in 2017 [30] investigated the synergistic combination of the advanced material properties offered by sol–gel graphene sorbent and the simplicity of the fabric phase sorptive extraction approach in selectively extracting bisphenol A and residual monomers including bisphenol A glycerolatedimethacrylate, urethane dimethacrylate, and triethylene glycol dimethacrylate-derived dental restorative materials from cow and human breast milk samples. After evaluation of the extraction efficiency of different coatings, sol–gel graphene coated media proved to show the best results. The main experimental parameters affecting the analytes’ extraction, namely: sorbent chemistry used, sample loading conditions, elution solvent, sorption stirring time, elution time, impact of protein precipitation, amount of sample, and matrix effect, were investigated and optimized. Absolute recovery values from standard solutions were 50% for bisphenol A, 78% for triethylene glycol dimethacrylate, 110% for urethane dimethacrylate, and 103% for bisphenol A glycerolatedimethacrylate, while respective absolute recovery values from milk were 30%, 52%, 104%, and 42%. The developed method was validated according to European Decision 657/2002/EC.

Aznar et al. in 2017 [31] proposed a simple, fast, and sensitive analyte extraction method based on fabric phase sorptive extraction (FPSE) followed by gas chromatography mass spectrometry (GC-MS) and ultra-performance liquid chromatography-quadrupole time of flight mass spectrometry (UPLC-QTOF-MS) analysis for the analysis of 12 volatile and semi-volatile compounds, namely: furfuryl alcohol, butyric acid, cis-3-hexen-1-ol, ethyl butyrate, vanillin, ethyl isovalerate, linalool, 1-octen-3-one, eugenol, octanal, ethyl octanoate, and limonene, which represent most of the principal chemical families possessing different polarities and volatilities. Five FPSE membranes coated with different sol–gel sorbent chemistries having different polarities and selectivities were evaluated: long chain poly(dimethylsiloxane) (sol–gel PDMS), short chain poly(tetrahydrofuran) (sol–gel PTHF), Carbowax 20M (sol–gel CW20M), short chain poly(dimethyl siloxane) (sol–gel SC PDMS), and polyethylene glycol-polypropylene glycol-polyethylene glycol triblock copolymer (sol–gel PEG-PPG-PEG). Sol–gel CW20M coated FPSE media showed the best extraction performance. The developed methodology was applied to the analysis of orange juice obtained from fresh oranges and oranges after storing at 5 °C for two months in order to identify the best chemical markers, both volatiles and non-volatiles, attributed to the freshness of the orange.

Santana Viera et al. in 2017 [32] developed an FPSE based method for the analysis of seven cytostatic drug compounds that are commonly used in anti-cancer therapies prior to ultra-high-performance liquid chromatography tandem mass spectrometry (UHPLC-MS/MS). The extraction protocol was optimized after investigation of the major parameters that affect the extraction efficiency. The detection limit of the method was within the values at which these compounds are usually found in environmental water (0.20 ng L^−1^ to 80 ng L^−1^). The applicability of the method was proved by the analysis of real wastewater samples from an effluent obtained from a hospital area and three wastewater treatment plants located in Gran Canaria Island, Spain.

Sol–gel M-CW20M was the fabric media that achieved better results, with a range of absolute recoveries between 25% and 90% using methanol as the eluting solvent.

Yang et al. in 2018 [33] proposed a green, simple, inexpensive, and sensitive ionic liquid immobilized fabric phase sorptive extraction method coupled with high performance liquid chromatography for the rapid screening and simultaneous determination of four fungicides (azoxystrobin, chlorothalonil, cyprodinil, and trifloxystrobin) residues in tea infusions. The optimum conditions were found to be 10% [HIMIM]NTf_2_ as coating solution, 2 min vortex time, 500 μL acetonitrile as dispersive solvent, and 2 min desorption time. Under these conditions, the proposed technique was applied to detect fungicides from real tea water samples with satisfactory results.

Rekhi et al. in 2018 [34] reported on the determination of trace levels of aluminum by high-performance liquid chromatography (HPLC) with UV detection using quercetin, a natural bioactive flavonol, as a metal complexation agent. The developed method has been successfully applied to the direct determination of aluminum in water samples derived from various sources. Fabric phase sorptive extraction (FPSE) was applied for the preconcentration of aluminum due to its presence in environmental water at trace levels. Efficient extraction of the quercetin-Al(III) complex from aqueous samples has been accomplished by applying FPSE using a cellulose fabric substrate coated with the sol–gel C18 hybrid nanocomposite sorbent.

Kabir et al. in 2018 [35] proposed a novel fabric phase sorptive extraction high-performance liquid chromatography-photodiode array detection (FPSE-HPLC-PDA) method for the simultaneous extraction and analysis of three drug residues (ciprofloxacin, sulfasalazine, and cortisone) in human whole blood, plasma, and urine samples, which are generally administered in human patients to treat inflammatory bowel disease (IBD). The analytical method was optimized and validated in the range 0.05–10 μg/mL for whole blood, 0.25–10 μg/mL for human plasma, and 0.10–10 μg/mL for human urine. The performance of the validated FPSE-HPLC-PDA was proved on real IBD patient samples. The developed method was shown to be a rapid, robust, and green analytical tool for clinical and pharmaceutical applications.

Sol–gel CW 20M media were found to yield the best recoveries using methanol for back extraction. The FPSE membrane can be reused up to approximately 30 times when washed by 2 mL acetonitrile: methanol (50:50, *v*:*v*) for 5 min and subsequently dried and stored in a hermetically sealed glass manifold, with no appreciable carry-over and no efficiency loss.

In 2018, Kazantzi et al. [36] proposed an automatic sample preparation (preconcentration/separation) based on a novel sol–gel sorbent based on caprolactone-dimethylsiloxane-caprolactone block polymer comprised of a nonpolar dimethylsiloxane and hydrophilic caprolactone as a coating on hydrophobic polyester fabric substrate and investigated its evaluation in an automatic FDSE on-line fabric disk sorptive extraction (FDSE) system coupled with flame atomic absorption spectrometry (FAAS). The proposed flow injection system was evaluated for the analysis of trace Cu(II), Ni(II), Zn(II), Pb(II), and Cd(II) in urine samples. The method was based on the on-line formation of target analytes with ammonium pyrrolidine dithiocarbamate (APDC) and their retention onto the surface of the fabric disk medium. Methyl isobutyl ketone (MIBK) was used to elute metal-APDC complexes directly into the nebulizer–burner system of FAAS. For 90 s of preconcentration time, enhancement factors of 250, 130, 185, and 36 and detection limits (3 s) of 0.15, 0.41, 1.62, and 0.49 μg L^−1^ were obtained for Cu(II), Ni(II), Pb(II), and Cd(II), respectively. For 30 s of preconcentration time, an enhancement factor of 49 and a detection limit of 0.12 μg L^−1^ were achieved for Zn(II) determination. The method was tested by analyzing certified reference materials and biological samples. 

Locatelli et al. in 2018 [37] described a fast, sensitive, and selective procedure for the analysis of aromatase inhibitors including anastrozole, letrozole, and exemestane used in the treatment of metastatic breast cancer by high performance liquid chromatography (HPLC) in human whole blood, plasma, and urine samples based on fabric phase sorptive extraction (FPSE). Validation was performed following the demands of international guidelines on bioanalytical methods validation. The analytical performance was proved on real human biological samples. The developed protocol can be readily applied for clinical and pharmaceutical analyses.

Six different FPSE membrane chemistries were primarily evaluated: sol–gel CW 20M, sol–gel PEG-PPG-PEG, sol–gel PCAP-PDMS-PCAP, sol–gel octadecyl (C18), sol–gel polycaprolactone A, and sol–gel sucrose. Three of these membranes performed better for the extraction of the examined analytes: sol–gel CW 20M, sol–gel PEG-PPG-PEG, and sol–gel polycaprolactone using methanol for back extraction.

Kaur et al. in 2019 [11] combined FPSE with gas chromatography mass spectrometry for the rapid extraction and determination of nineteen organochlorine pesticides in various fruit juices and water samples. The extraction approach was optimized in terms of sorbent chemistry, extraction time, stirring speed, type and volume of back-extraction solvent, and back-extraction time. Optimum conditions yielded limits of detection in a range of 0.007–0.032 ng/mL. The relative recoveries obtained by spiking organochlorine pesticides in water and selected juice samples were in the range of 91.56–99.83%. Sol–gel poly(ethylene glycol)-poly(propylene glycol)-poly(ethylene glycol) was proved to be the best sorbent for the extraction and preconcentration of organochlorine pesticides in aqueous and fruit juice samples prior to analysis with gas chromatography mass spectrometry. 

Tartaglia et al. in 2019 [38] reported on the performance comparison between the exhaustive and equilibrium extraction using classical Avantor C18 solid phase extraction (SPE) sorbent, hydrophilic-lipophilic balance (HLB) SPE sorbent, Sep-Pak C18 SPE sorbent, novel sol–gel Carbowax 20M (sol–gel CW 20M) SPE sorbent, and sol–gel CW 20M coated fabric phase sorptive extraction (FPSE) media for the extraction of three inflammatory bowel disease (IBD) drugs. Both the commercial SPE phases and in-house synthesized sol–gel CW 20M SPE phases were loaded into SPE cartridges and the extractions were carried out under an exhaustive extraction mode, while FPSE was carried out under an equilibrium extraction mode. The method was validated in compliance with international guidelines for the bioanalytical method validation. Novel in-house synthesized and loaded sol–gel CW 20M SPE sorbent cartridges were characterized in terms of their extraction capability, breakthrough volume, retention volume, hold-up volume, number of the theoretical plate, and the retention factor.

The performance of FPSE and SPE techniques was evaluated by comparing the breakthrough volume and enrichment factors. The authors found that for the examined analytes, SPE showed the highest enrichment factors; consequently, this method is more suitable for samples with low analytes concentration.

Perez Mayan in 2019 [39] investigated the use of FPSE for the extraction and preconcentration of ultra-trace level residues of fungicides (19 compounds) and insecticides (3 species) in wine samples. Subsequently, the preconcentrated analytes were determined using ultra-performance liquid chromatography–tandem mass spectrometry (UPLC-MS/MS). Experimental extraction parameters affecting the efficiency and repeatability of the extraction were optimized. Optimized conditions included cellulose fabric coated with a sol–gel polyethylene glycol sorbent and back extraction using ACN-MeOH (80:20 *v*/*v*) mixture. Limits of quantification (LOQs) ranged between 0.03 and 0.3 ng mL^−1^. Relative recoveries ranged from 77 ± 6% to 118 ± 4%, and from 87 ± 4% to 121 ± 6% for red and white wines, respectively. The applicability of the method was proved for commercial wines.

In 2019, Lioupi et al. [40] developed and validated an innovative fabric phase sorptive extraction high-performance liquid chromatography–diode array detection (FPSE-HPLC-DAD) method for the extraction of five common antidepressants (venlafaxine, paroxetine, fluoxetine, amitriptyline, clomipramine) in human urine samples. The extraction protocol was optimized with regards to the extraction main parameters. Sol–gel graphene sorbent, coated on cellulose FPSE media, were the most efficient among other with different polarities using CH_3_OH:CH_3_CN (50:50 *v*/*v*) for back-extraction. The absolute recovery values were 25.5% for venlafaxine, 33.9% for paroxetine, 67.0% for fluoxetine, 43.0% for amitriptyline, and 29.0% for clomipramine, while relative recoveries were higher than 90%. The developed method provides satisfactory limit of detection 0.15 ng/μL.

Locatelli in 2019 [41] proposed a fabric phase sorptive extraction based method prior to high-performance liquid chromatography–photodiode array detection (FPSE-HPLC-PDA) for the simultaneous extraction and analysis of six benzophenone derivative UV filters, including benzophenone (BZ), 5-benzoyl-4-hydroxy-methoxybenzenesulfonic acid (BP-4), bis(4-hydroxyphenyl)methanone (4-DHB), bis(2,4-dihydroxyphenyl)methanone (BP-2), (2,4-dihydroxybenzophenone) (BP-1), and 2,2′-dihydroxy-4-methoxybenzophenone (DHMB) in human whole blood, plasma, and urine samples. The limit of quantification was found to be 0.1 μg/mL. This new approach shows promising results with high potential for direct adaptation as a rapid, robust, and green analytical tool for several applications, e.g., in the current sample preparation practices used in many bioanalytical fields including pharmacokinetics (PK), pharmacodynamics (PD), therapeutic drug monitoring (TDM), clinical and forensic toxicology, disease diagnosis, and drug discovery. Optimized conditions included the use of sol–gel CW^®^20M FPSE membrane with a 20:80 (% *v:v*) mixture of phosphate buffer 40 mM at pH 3 and methanol.

Taraboletti et al. in 2019 [42] reported a metabolomics workflow using a mass spectrometry-compatible fabric phase sorptive extraction (FPSE) technique implementing a matrix coated with sol–gel poly(caprolactone-b-dimethylsiloxane-b-caprolactone) that binds both polar and nonpolar metabolites in whole blood, eliminating serum processing steps. FPSE preparation technique combined with liquid chromatography–mass spectrometry can distinguish radiation exposure markers such as taurine, carnitine, arachidonic acid, α-linolenic acid, and oleic acid found 24 h after 8 Gy irradiation. These findings suggest that the FPSE approach could work in future technology to triage irradiated individuals accurately, via biomarker screening, by providing a novel method to stabilize biofluids between collection and sample analysis.

Alampanos et al. in 2019 [43] proposed an environmentally friendly method by making use of high-performance liquid chromatography and photo-diode array detection (HPLC-PDA) for the determination of four penicillin antibiotics residues (benzylpenicillin, cloxacillin, dicloxacillin, and oxacillin) in human blood serum after FPSE. Solvent evaporation and reconstitution steps, which are considered to be rather time-consuming, were eradicated successfully from the sample preparation workflow, organic solvent consumption was brought to a minimum, while protein precipitation was assessed as impractical. Thus, the proposed method met all green analytical chemistry (GAC) criteria. The microextraction device was characterized by high chemical and solvent stability owing to the strong chemical bonds formed between the sol–gel sorbent and the substrate. Therefore, any organic solvent/solvent mixture can serve as the eluent/back-extraction solvent. The authors, after optimization of FPSE experimental parameters, propose sol–gel poly(tetrahydrofuran) coated FPSE membrane as the optimum extraction sensitivity for the selected penicillin antibiotics, after back-extraction using 90:10 *v*/*v* acetonitrile and ammonium acetate (0.01M). For all four penicillin antibiotics, the limit of detection was 0.15 ng/μL.

Zilfidou et al. in 2019 [44] applied FPSE for the simple and rapid simultaneous extraction of five common antidepressant drug residues (venlafaxine, paroxetine, fluoxetine, amitriptyline, and clomipramine) from human blood serum. Elimination of protein precipitation step and minimized solvent consumption led to a sample preparation workflow compliant with the principles of green analytical chemistry (GAC). Among all the membrane examined, sol–gel polycaprolactone-dimethylsiloxane-polycaprolactone coated polyester substrate presented optimum extraction efficiency and was found to be reusable for at least 30 times. Back-extraction was achieved by methanol: acetonitrile (50:50 *v*/*v*). The limit of detection was found at 0.15 ng μL^−1^, while good absolute recoveries (9.4–88.1%) were obtained.

Tartaglia et al. in 2019 [45] described an FPSE based method for the simultaneous determination of seven paraben residues including methyl paraben (MPB), ethyl paraben (EPB), propyl paraben (PPB), isopropyl paraben (iPPB), butyl paraben (BPB), isobutyl paraben (iBPB), and benzyl paraben (BzPB) in human whole blood, plasma and urine, prior to high-performance liquid chromatography (HPLC) coupled with photo diode array detector (PDA) analysis. The analytical method has been validated according to the international guidelines.

The performance of the analytical method was evaluated on real biological samples. The proposed innovative method allows simultaneous analysis of seven paraben residues in three different biological matrices, including whole blood, plasma, and urine, and therefore it is easily applicable to monitor these substances in different biological samples. Furthermore, the extraction technique used in this work is fast, easy to use, and in accordance with the modern green analytical chemistry (GAC) principles. Sol–gel CW 20M FPSE media and back-extraction with methanol provided the best recovery rates.

Kaur et al. in 2019 [11] combined FPSE with gas chromatography–mass spectrometry for the rapid extraction and determination of nineteen organochlorine pesticides in various fruit juices and water samples. FPSE efficiency was optimized in terms of sorbent chemistry, extraction time, stirring speed, type and volume of back-extraction solvent, and back-extraction time. Under optimum conditions, the limits of detection were obtained in a range of 0.007–0.032 ng/mL. The relative recoveries obtained by spiking organochlorine pesticides in water and selected juice samples were in the range of 91.56–99.83%. The sorbent sol–gel poly(ethylene glycol)-poly(propylene glycol)-poly(ethylene glycol) was applied for the extraction and preconcentration of organochlorine pesticides in aqueous and fruit juice samples prior to analysis with gas chromatography–mass spectrometry.

Otoukesh et al. in 2019 [46] proposed a fabric phase sorptive extraction (FPSE) for the enrichment of acrylate compounds coming from acrylic adhesives used commonly for sticking the paper labels on polyethylene terephthalate (PET) bottles, and therefore they may exist in recycled polyethylene terephthalate (rPET) in different food simulants: simulant A (ethanol 10%), simulant B (acetic acid 3%), and simulant C (ethanol 20%), and their respective extracts by ultra-high-performance liquid chromatography with mass spectrometric detection (UPLC-MS). Four acrylates were studied: ethylene glycol dimethacrylate (EGDM), pentaerythritol triacrylate (PETA), triethylene glycol diacrylate (TEGDA), and trimethylolpropane triacrylate (TMPTA). Five different types of FPSE membrane coated with different sol–gel sorbents were studied, and finally sol–gel polyethylene glycol- polypropylene glycol-polyethylene glycol triblock copolymer (PEG-PPG-PEG) coated FPSE membrane was chosen for its satisfactory results combined with methanol for back-extraction since it provided an elution ability slightly higher than acetonitrile. Under the optimized conditions, the method provided limits of detection of the compounds in the range of (0.1–1.9 ng g^−1^, 0.1–1.2 ng g^−1^, 0.2–2.3 ng g^−1^) in EtOH 10%, HAc 3%, and EtOH 20%, and the enrichment factor values (EFs) after applying N_2_ were in the range of 11.1–25.0, 13.8–26.3, 8.3–21.9, in simulants A, B, and C, respectively. The optimized method was applied successfully to analyze thirteen types of recycled PET samples.

Mesa et al. in 2019 [10] developed a simple and sensitive analytical methodology for rapid screening and quantification of selected estrogenic endocrine disrupting chemicals including α-estradiol, hexestrol, estrone, 17α-ethinyl estradiol, diethylstilbestrol, and bisphenol A from intact milk using fabric phase sorptive extraction in combination with high-performance liquid chromatography coupled to ultraviolet detection/tandem mass spectrometry. The new approach eliminates protein precipitation and defatting step from the sample preparation workflow, while the error prone and time-consuming solvent evaporation and sample reconstitution steps have also been eliminated. Parameters which mostly affect the extraction efficiency of fabric phase sorptive extraction, including sorbent chemistry, sample volume, and extraction time, were optimized. The limit of detection values obtained in fabric phase sorptive extraction with high-performance liquid chromatography with ultraviolet detection ranged from 25.0 to 50.0 ng/mL.

Two sol–gel sorbent coatings were tested to determine the better sorbent coating for the selected EDCs, sol–gel PTHF, and sol–gel PDMS. Sol–gel PTHF was distinctly superior in extraction efficiencies for all compounds, with acetonitrile used for back extraction.

Lastovka et al. in 2019 [47] developed a method for the quantification of highly potent analgesic agent (2*R*,4a*R*,7*R*,8a*R*)-4,7-dimethyl-2-(thiophen-2-yl)octahydro-2*H*-chromen-4-ol in rat whole blood and plasma using dried matrix spots (DMS) and fabric phase sorptive extraction (FPSE) techniques in combination with LC–MS/MS. The linearity was obtained in the range of 20–5000 ng/mL and 50–5000 ng/mL for plasma-FPSE and blood-FPSE experiments, respectively. The mean extraction recovery (%) was 26 for plasma-DMS, 25 for blood DMS, 38 for plasma-FPSE, and 31 for blood-FPSE.

A sol–gel PCAP-PDMS-PCAP sorbent-coated FPSE biofluid sampler FPSE blood sampler was compared to a DBS card and has been used under a different sampling and extraction mode (DBS card with direct spotting, and FPSE biofluid sampler with equilibrium extraction mode); both perform satisfactorily with different sample matrices. However, the FPSE biofluid sampler was found more selective in preparing an interferents-free sample for instrumental analysis. Due to the exploitation of high-performance sol–gel sorbent, the FPSE biofluid sampler has the potential to streamline the current practice of blood analysis.

Gulle et al. in 2019 [48] developed a fabric phase sorptive extraction (FPSE)-based sample preparation method for methyl paraben (MP), propyl paraben (PP), and butyl paraben (BP) in cosmetic and environmental samples, prior to high performance liquid chromatography–photodiode array (HPLC-PDA) detection. In the proposed method, MP, PP, and BP molecules were efficiently retained on a sol–gel Carbowax-20M sorbent-coated FPSE membrane when the matrix pH was adjusted to 5. Subsequently, the extracted analytes were desorbed from the FPSE membrane with methanol. Experimental conditions were studied to optimize variables such as pH, adsorption time, and desorption solvent. Using the optimal conditions, analytical parameters such as linearity ranges, detection limits, and preconcentration factors for each of the selected parabens were calculated from experimental data. The limit of detection (LOD) values for MP, PP, and BP were calculated as 2.85, 2.98, and 2.75 ng mL^−1^, respectively. Finally, the developed method was applied to cosmetic and environmental samples.

Kaur et al. in 2019 [49] developed a high-efficiency and solvent minimized microextraction technique, fabric phase sorptive extraction followed by gas chromatography and mass spectrometry analysis for the rapid determination of four organophosphorus pesticides (terbufos, malathion, chlorpyrifos, and triazofos) in vegetable samples including beans, tomato, brinjal, and cabbage. The most important fabric phase sorptive extraction parameters were investigated and optimized. Under optimum experimental conditions, the limits of detection were found in the range of 0.033 to 0.136 ng/g. Three different sol–gel sorbent coated FPSE membranes were evaluated, including sol–gel Carbowax 20 M (sol–gel CW 20 M), sol–gel poly(tetrahydrofuran) (sol–gel PTHF), and sol–gel poly(dimethyl siloxane) (sol–gel PDMS). Sol–gel CW 20 M coated FPSE membrane was selected as the suitable FPSE membrane for the selected OPPs.

Sun et al. in 2019 [50] developed a new method which coupled FPSE with ion mobility spectrometry (IMS) for the rapid detection of polycyclic aromatic hydrocarbons (PAHs) in water present in the field. Polydimethylsiloxane (PDMS) was coated on the glass fiber cloth through a sol–gel reaction. After extracting the PAHs in water, the fabric coated PDMS could be directly put into the inlet of IMS instrument for thermal desorption. The PAHs were analyzed by the IMS instrument operated in the positive ion mode with a corona discharge (CD) ionization source. The primary parameters affecting extraction efficiency such as extraction time, extraction temperature, and ionic strength were investigated and optimized by using phenanthrene (Phe), benzo[a]anthracene (BaA), and benzo[a]pyrene (BaP) as model compounds. Under the optimal conditions, the FPSE-IMS detection limits were 5 ng mL^−1^, 8 ng mL^−1^, and 10 ng mL^−1^, respectively. Satisfactory recoveries were obtained ranging from 80.5% to 100.5%, making the method of FPSE-IMS applicable for the monitoring the water quality on-site, and thus providing early warning in the field.

Kaur et al. in 2019 [51] developed and validated a rapid extraction and clean-up method using selective fabric phase sorptive extraction combined with gas chromatography and mass spectrometry for the determination of broad polarity spectrum emerging pollutants, ethyl paraben, butyl paraben, diethyl phthalate, dibutyl phthalate, lidocaine, prilocaine, triclosan, and bisphenol A in various aqueous samples. Some important parameters of fabric phase sorptive extraction such as extraction time, matrix pH, stirring speed, type, and volume of desorption solvent were investigated and optimized. Under the optimum conditions, the limits of detection were in the range 0.009–0.021 ng/mL. Recoveries ranged from 93 to 99%. The developed method was applied for the determination of the emerging contaminants in tap water, municipal water, ground water, sewage water, and sludge water samples. Three different FPSE sorbent coatings were comparatively studied: sol–gel CW20M (polar), sol–gel PTHF (medium polar), and sol–gel C18 (nonpolar). Sol–gel CW20M-coated FPSE provided the optimum results.

The most recent application comes from Celeiro et al. [52]. This research group in 2020 proposed a novel method based on fabric phase sorptive extraction (FPSE) followed by gas chromatography–tandem mass spectrometry (GC-MS/MS) for the simultaneous determination of 11 UV filters (ethylhexyl salicylate, benzyl salicylate, homosalate, benzophenone-3, isoamylmethoxycinnamate, 4-methylbenzylidenecamphor, methyl anthranilate, etocrylene, 2-ethylhexylmethoxycinnamate, 2-ethylhexyl p-dimethylaminobenzoate, and octocrylene), in natural and recreational waters. Different types and sizes of sol–gel coated FPSE membranes, sample volumes, extraction times, and types and volumes of desorption solvent were optimized. The optimal conditions involved the use of a (2.0 × 2.5) cm^2^ FPSE device with PDMS-based coating for the extraction of 20 mL of water for 20 min. Back-extraction was performed by ethyl acetate. Recovery rates under optimum conditions were about 90%. LODs were at the low ng L^−1^ in all cases. The proposed validated FPSE-GC-MS/MS method was applied to different real samples, including environmental water (lake, river, seawater) and recreational water (swimming pool).

## 8. Trend and Future Perspectives

**Figure 3:**
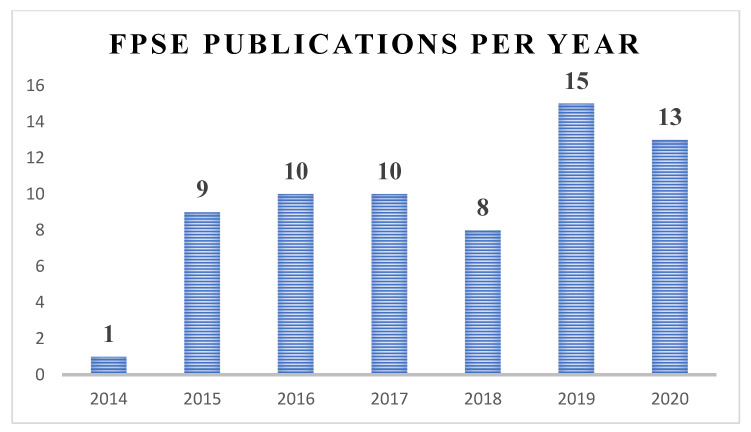
Graphical representation of number of papers published on FPSE per year since 2014.

**Figure 4:**
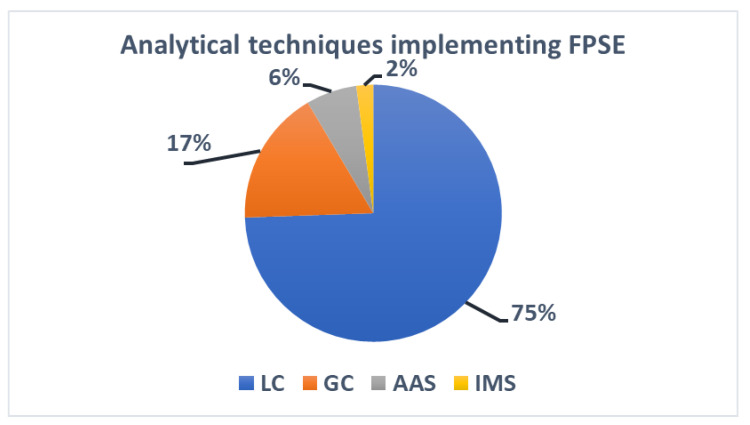
Graphical representation of different analytical instruments used (%) subsequent to FPSE.

As it is shown in Figure 3, publications based on FPSE constantly increased, and it can be predicted that it will expand to more analytes as well as more sample matrices in the future. However, there is a slight decrease in the number of published papers in 2020, which may be attributed to the global pandemic that slowed down everything, including scientific research. Among the published applications, the vast majority use liquid chromatographic determination with various detection and identification techniques (Figure 4). However, this tendency has the potential to be altered in the future, since more applications are anticipated covering all chemistries of analytes. New sorptive membranes in new formats can help this direction. Additionally, the ability for implementation in automated systems meets the new analytical performance criteria, and more on-line approaches are expected to be developed.

## 9. Conclusions

Fabric phase sorptive extraction has emerged as a new generation sample preparation technique with many new attributes that were not offered before by a single extraction/microextraction technique. Although FPSE is not commercially available yet, it has successfully established itself as an inevitable laboratory consumable within a short period. Many academic research groups across the world have demonstrated the performance superiority, compliance of green analytical principles, substantial minimization of sample preparation workflow, extended pH working range, reusability, and field deployability of FPSE membranes in numerous applications using diversified sample matrices which will undoubtedly provoke new analysts to explore this powerful technology. A broad range of sorbents chemistries offered by FPSE encompassing all the sorbent chemistries available on the SPE and SPME platform will provide an analyst more liberty to select the appropriate sorbent for a given application. Ability to use the same FPSE membrane in SPME mode or SPE mode is indeed a unique concept in the rapidly growing sample preparation technology space. The mathematical model-driven sorbent selection strategy proposed by FPSE also manifests another green component that was not considered before and deserves appreciation.

## Figures and Tables

**Table 1:** List of major fabric phase sorptive extraction (FPSE) sorbents.

Name of the SorbeSorbent Coating	Polarity of the Sorbent
Neutral Sorbents	
1. Sol–gel poly(dimethylsiloxane)	Nonpolar
2. Sol–gel poly(dimethyldiphenylsiloxane)	Nonpolar
3. Sol–gel methyl	Nonpolar
4. Sol–gel C4	Nonpolar
5. Sol–gel C8	Nonpolar
6. Sol–gel C12	Nonpolar
7. Sol–gel C18	Nonpolar
8. Sol–gel Graphene	Nonpolar
9. Sol–gel Multi Wall Carbon Nanotubes	Nonpolar
10. Sol–gel Single Wall Carbon Nanotubes	Nonpolar
11. Sol–gel Activated Carbon	Nonpolar
12. Sol–gel poly(tetrahydrofuran)	Medium polar
13. Sol–gel poly(ethylene glycol)-poly(propylene glycol)-poly(ethylene glycol)	Medium polar
14. Sol–gel poly(propylene glycol)-poly(ethylene glycol)-poly(propylene glycol)	Medium polar
15. Sol–gel propyl methacrylate	Medium polar
16. Sol–gel poly(caprolactone)-poly(dimethylsiloxane)-poly(caprolactone	Medium polar
17. Sol–gel poly(caprolactone)-poy(tetrahydrofuran)-poly(caprolactone)	Medium polar
18. Sol–gel poly(caprolactone diol)	Medium polar
19. Sol–gel poly(caprolactone triol)	Medium polar
20. Sol–gel Silica	Polar
21. Sol–gel Sucrose	Polar
22. Sol–gel Sucralose	Polar
23. Sol–gel Chitosan	Polar
24. Sol–gel Carbowax 20M	Polar
25. Sol–gel poly(ethylene glycol), 300	Polar
26. Sol–gel poly(ethylene glycol), 10,000	Polar
**Mixed Mode Sorbents**	
27. Sol–gel cation exchanger, C18	Ion exchanger/nonpolar
28. Sol–gel anion exchanger, C18	Ion exchanger/nonpolar
29. Sol–gel Zwitterionic cation exchanger, anion exchanger, C18	Dual ion exchanger/nonpolar
**Ion Exchanger Sorbents**	
30. Sol–gel cation exchanger	Ion exchanger
31. Sol–gel anion exchanger	Ion exchanger
32. Sol–gel Zwitterionic anion and cation exchanger	Ion exchanger

**Table 2:** Absolute recovery calculator for selected FPSE membranes.

Sorbent (Substrate)	Equation for Recovery% Calculation
Si-CW20M (Cellulose)	4.2977487 + 22.823041 log Kow − 3.1343544 (log Kow − 2.737)^2^
Si-PEG1000 (Cellulose)	−11.53483 + 20.950137 log Kow − 0.4017218 (log Kow − 2.737)^2^
Si-PEG300 (Cellulose)	14.758805 + 16.309632 log Kow − 5.5504622 (log Kow − 2.737)^2^
Si-CN-CW20M (Cellulose)	−24.39275 + 23.940499 log Kow + 1.247171 (log Kow − 2.737)^2^
Si-PPG-PEG-PPG (Cellulose)	−3.648816 + 21.546191 log Kow − 2.878525 (log Kow − 2.737)^2^
Si-PEG-PPG-PEG (Cellulose)	−7.680093 + 23.069108 log Kow − 1.7262745 (log Kow − 2.737)^2^
Si-PTHF (Cellulose)	12.40054 + 17.848979 log Kow + 17.848979 (log Kow − 2.737)^2^
Si-PTHF (Fiber Glass)	−28.44237 + 20.9507 log Kow + 3.3273496 (log Kow − 2.737)^2^
Si-C18 (Cellulose)	−2.274875 + 20.816015 log Kow − 4.1478973 (log Kow − 2.737)^2^
Si-C8 (Cellulose)	−3.392783 + 21.261305 log Kow − 3.7724155 (log Kow − 2.737)^2^
Si-PDPS (Cellulose)	−10.30009 + 17.450029 log Kow − 0.2880039 (log Kow − 2.737)^2^
Si-PDMDPS (Polyester)	−9.185327 + 17.815515 log Kow − 1.9655752 (log Kow − 2.737)^2^
Si-PDMDPS (Cellulose)	−19.60225 + 15.453851 log Kow − 1.62186 (log Kow − 2.737)^2^

**Table 3:** Comparison of extraction recovery between different fabric substrates.

Sorbent	FA (%)	PIP (%)	PHE (%)	BDO (%)	4NT (%)	9AM (%)	NAP (%)	TMB (%)	TCL (%)	DES (%)
Sol–gel PTHF (Cellulose)	0	9.8	4.0	45.6	46.7	66.1	86.8	93.4	82.1	49.5
Sol–gel PTHF (Fiber Glass)	0	1.2	4.1	21.8	25.4	25.4	38.4	77.8	74.9	91.7
Sol–gel PDMDPS (Polyester)	0	1.7	0	13.4	13.7	67.7	51.8	83.5	74.9	46.7
Sol–gel PDMDPS (Cellulose)	0	0.1	1.8	9.7	10.0	11.0	68.7	50.4	42.1	68.1
